# Supplementary material for: Solar-to-hydrogen peroxide energy conversion on resorcinol–formaldehyde resin photocatalysts prepared by acid-catalysed polycondensation
Source: Commun Chem. 2020 Nov 13;3:169. doi: 10.1038/s42004-020-00421-x (PMC9814707; doi:10.1038/s42004-020-00421-x)
Supplement: Supplementary file 1 — Supplementary Information [file 42004_2020_421_MOESM1_ESM.pdf]

Supplementary Online Material for

Solar-to-hydrogen peroxide energy conversion on resorcinol–formaldehyde resin photocatalysts prepared by acid-catalysed polycondensation

Yasuhiro Shiraishi,* Takumi Hagi, Masako Matsumoto, Shunsuke Tanaka,
Satoshi Ichikawa, and Takayuki Hirai

Supplementary Table 1 | Properties of RF resins prepared under different conditions[a] and their photocatalytic activity for H_2O_2 production.

entry	sample name	temperature / K	acid (or base)	resorcinol/formaldehyde/acid (or base) / mmol/mmol/mmol	pH	particle diameter / μm[b]	H_2O_2 / μmol[c,d]
1	RF-Na_2CO_3-523	523	Na_2CO_3	3.6/7.2/2.0	9.3		61.0
2	RF-NH_3-523	523	NH_3	3.6/7.2/3.0	7.8	0.57 ± 0.08	60.2
3	RF-$(COOH)_2$-523	523	$(COOH)_2$	3.6/7.2/0.18	3.0	3.09 ± 1.67	84.7
4	RF-HCl-523	523	HCl	3.6/7.2/0.15	3.1	3.10 ± 0.63	86.0
5	RF-H_2SO_4-523	523	H_2SO_4	3.6/7.2/0.11	3.1	2.54 ± 0.64	81.0
6	RF-HNO_3-523	523	HNO_3	3.6/7.2/0.16	3.0	2.52 ± 0.39	78.9
7	RF-CH_3COOH-523	523	CH_3COOH	3.6/7.2/0.50	3.8	2.58 ± 0.31	82.7
8		523		3.6/7.2/–	6.3	2.21 ± 0.87	56.0
9	RF-$(COOH)_2$-473	473	$(COOH)_2$	3.6/7.2/0.18	3.0	4.45 ± 1.29	69.1
10	RF-$(COOH)_2$-423	423	$(COOH)_2$	3.6/7.2/0.18	3.0	7.52 ± 1.08	39.8
11		373	$(COOH)_2$	3.6/7.2/0.18	3.0	no solid formed	
12		523	$(COOH)_2$	3.6/1.2/0.18	3.2		67.9
13		523	$(COOH)_2$	3.6/1.8/0.18	3.2		80.5
14		523	$(COOH)_2$	3.6/3.6/0.18	3.2		86.0
15		523	$(COOH)_2$	3.6/10.8/0.18	3.2		80.2
16		523	$(COOH)_2$	3.6/7.2/0.002	5.6		66.1
17		523	$(COOH)_2$	3.6/7.2/0.018	4.4		86.7
18		523	$(COOH)_2$	3.6/7.2/0.09	3.5		85.9
19		523	$(COOH)_2$	3.6/7.2/0.9	2.4	3.35 ± 1.23	91.0
20		523	$(COOH)_2$	3.6/7.2/1.8	1.7	3.90 ± 1.36	86.0

[a] All resins were prepared in water under hydrothermal conditions for 24 h.

[b] Determined by DLS.

[c] Reaction conditions: water (30 mL), catalyst (50 mg), O_2 (1 bar), λ >420 nm (Xe lamp, light intensity at 420−800 nm: 171.3 W m^{-2}), temperature (298 K), photoirradiation time (24 h).

[d] All of the data are the mean values determined by three independent experiments and contains ±6% deviations.

Supplementary Table 2 | The C=O and C‒O compositions of the RF resins determined by DD/MAS/^{13}C NMR and XPS analysis.

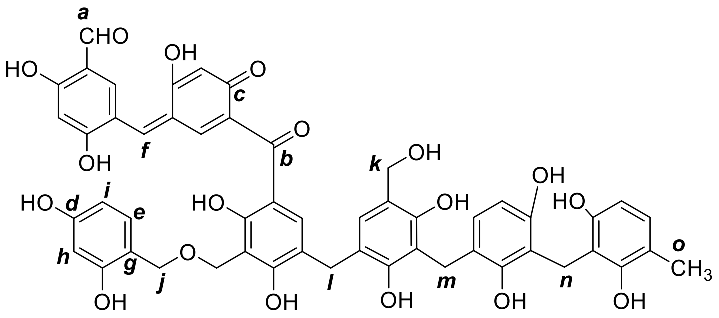

resins	DD/MAS/^{13}C NMR[a] / %		XPS (O 1s)[b] / %	
	C=O (***a***, ***b***, ***c***)[c]	C‒O (***d***, ***j***, ***k***)[c]	C=O	C‒O
RF-NH_3-523	28.3	71.7	28.2	71.8
RF-$(COOH)_2$-523	30.1	69.9	30.1	69.9
RF-$(COOH)_2$-523[d]	34.7	65.3	34.7	65.3
RF-$(COOH)_2$-473	28.8	71.2	29.0	71.0
RF-$(COOH)_2$-423	30.2	69.8	30.3	69.7

[a] The data are from Fig. 5 and Supplementary Fig. 7.

[b] The data are from Supplementary Fig. 9.

[c] Determined by sum of the peak areas for the respective carbon components shown in the round brackets.

[d] The RF-$(COOH)_2$-523 resin recovered after photoreaction for 5 h by a solar simulator (Fig. 4d).

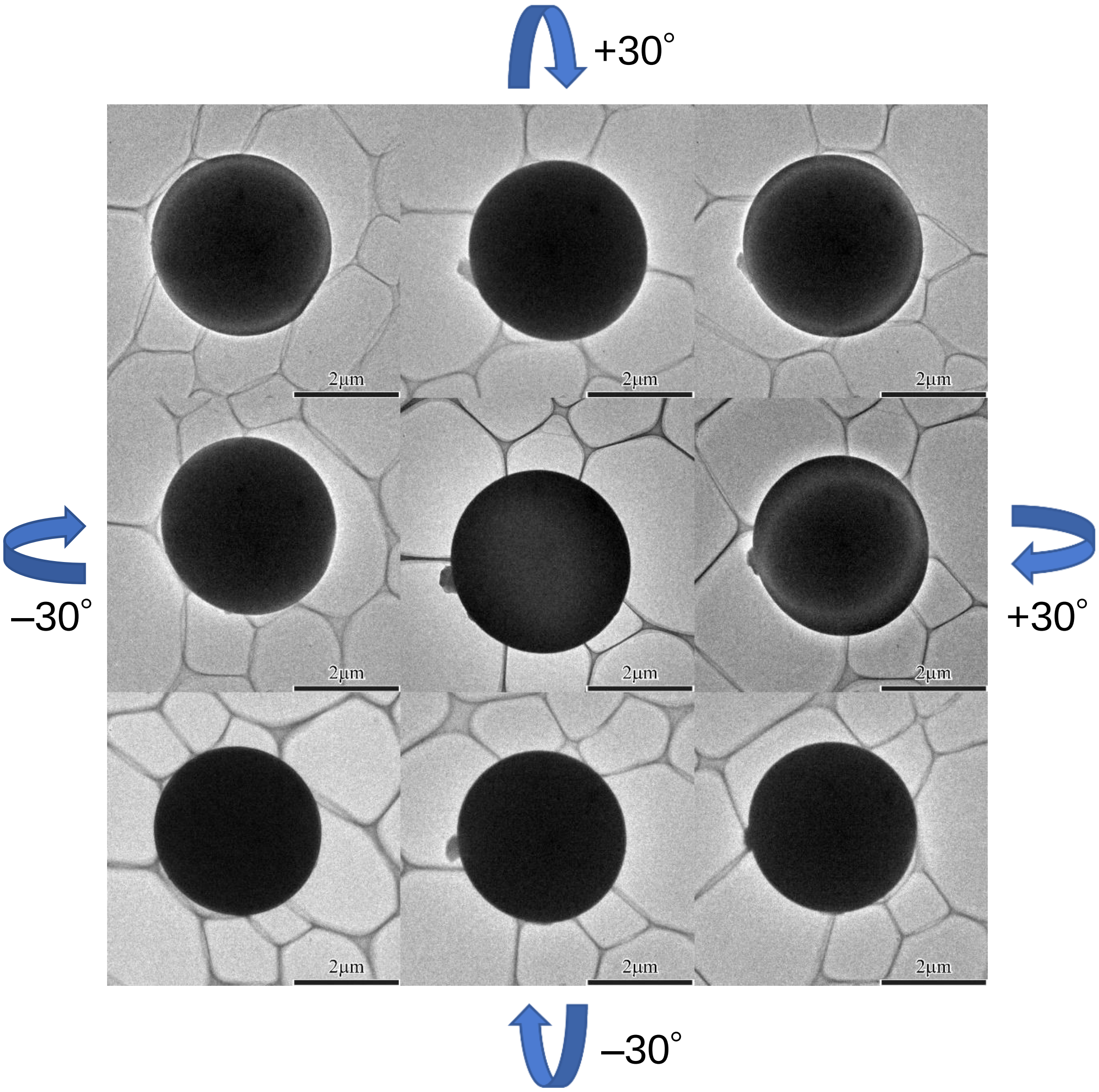

Supplementary Fig. 1 | TEM images of a RF-$(COOH)_2$-523 resin particle observed at different angles.

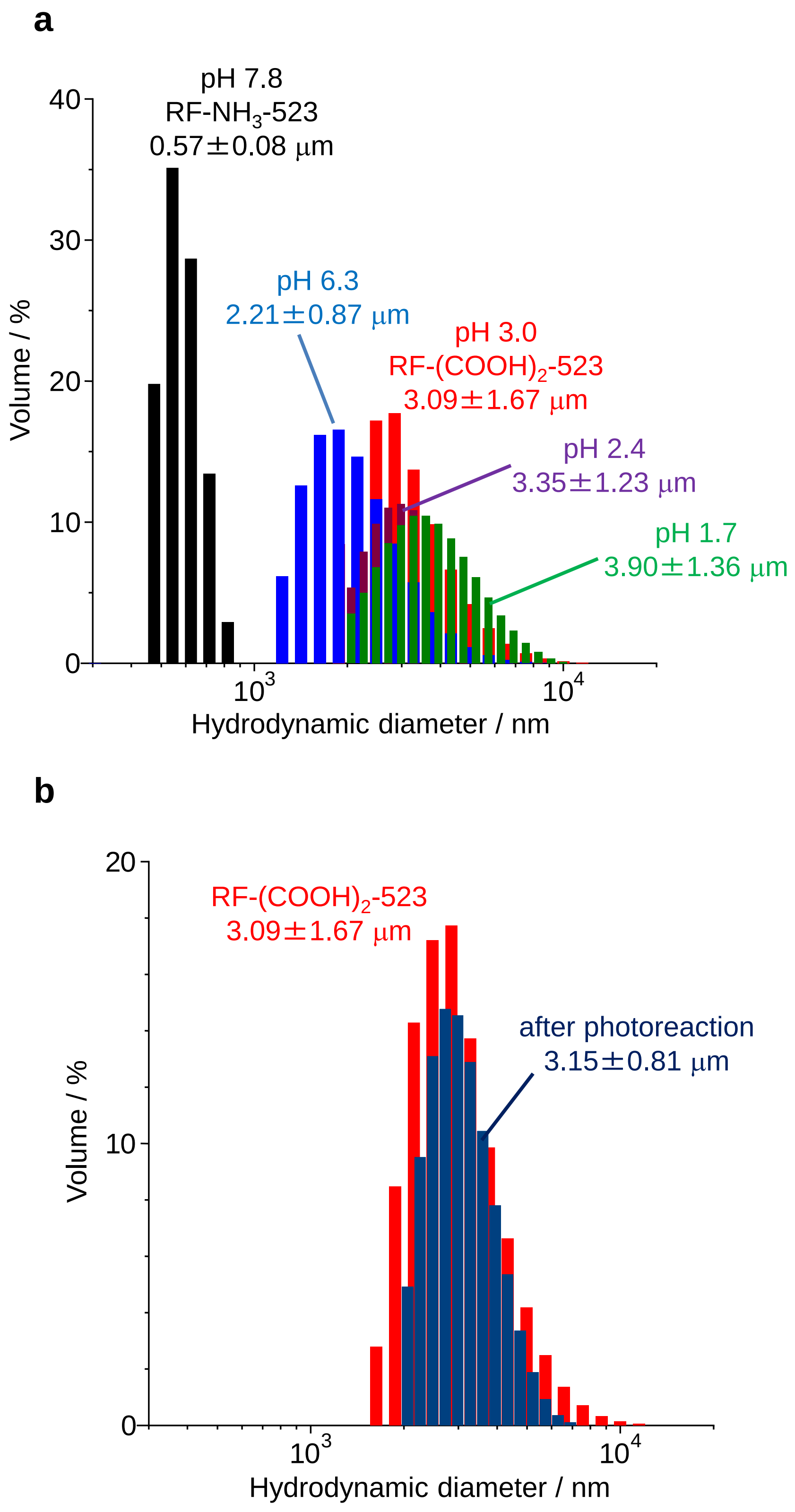

Supplementary Fig. 2 | DLS analysis of (a) the fresh resins prepared at different pH and (b) the RF-$(COOH)_2$-523 resin after photoreaction for 5 h by a solar simulator for 5 h (Fig. 4d). All resins were prepared at 523 K. In (a), the pH 6.3 sample was prepared in pure water, and the pH 2.4 and 1.7 samples were prepared by changing the amounts of $(COOH)_2$ added (see Supplementary Table 1).

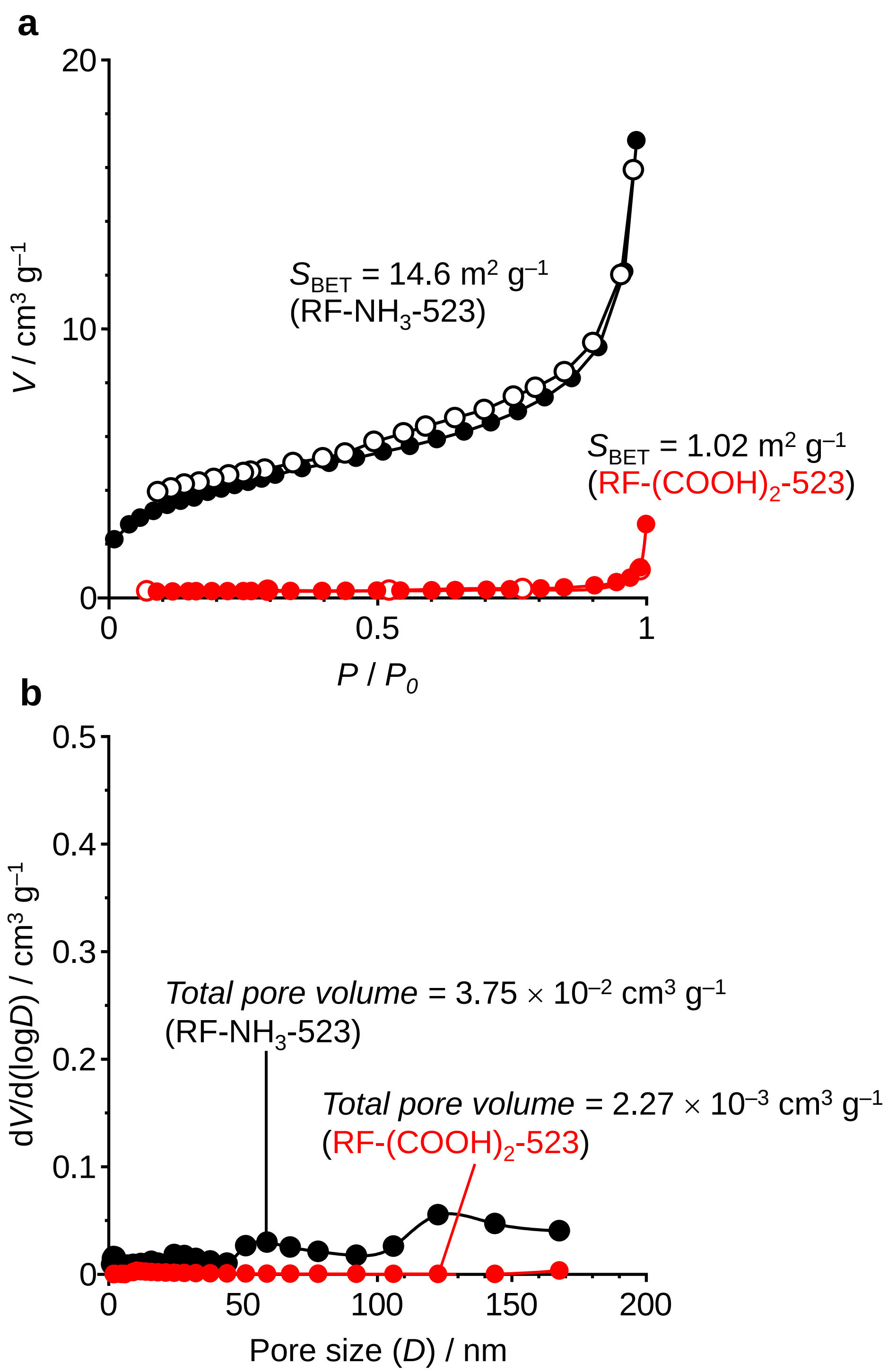

Supplementary Fig. 3 | (a) N_2 adsorption/desorption isotherms of the resins and (b) their pore size distributions determined by the BJH method.

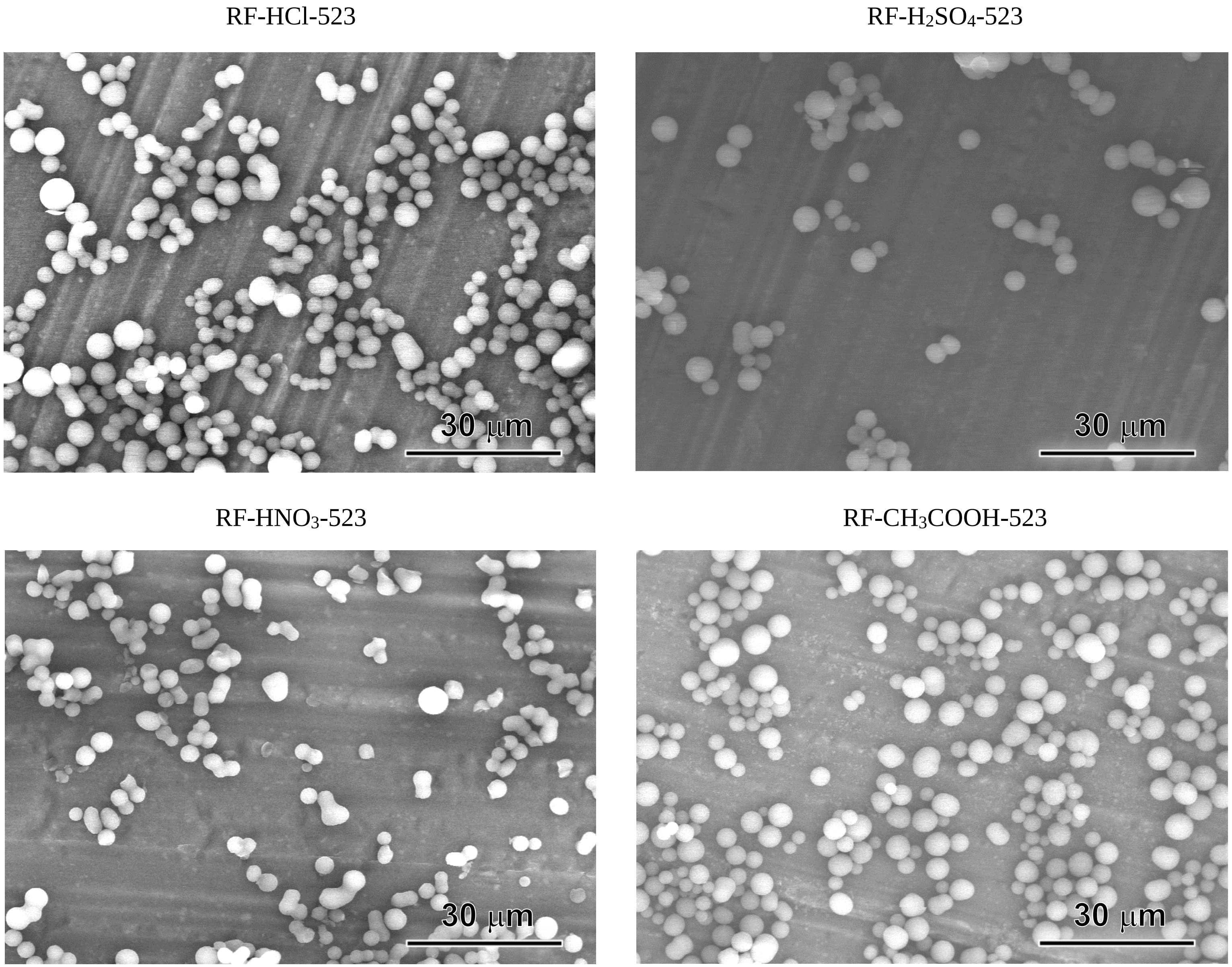

Supplementary Fig. 4 | SEM images of RF-acid-523 resins. The size of the particles determined by DLS is summarized in Supplementary Table 1.

pH 7.8 (RF-NH_3-573)

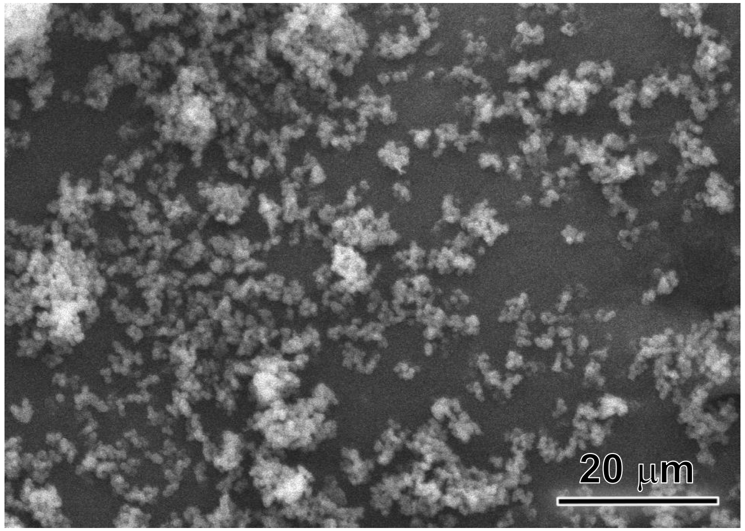

pH 6.3 (prepared in pure water)

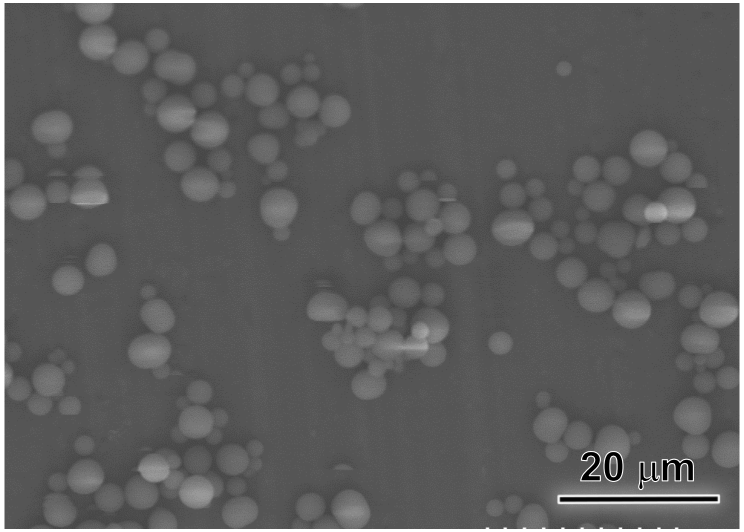

pH 2.4 (prepared with $(COOH)_2$)

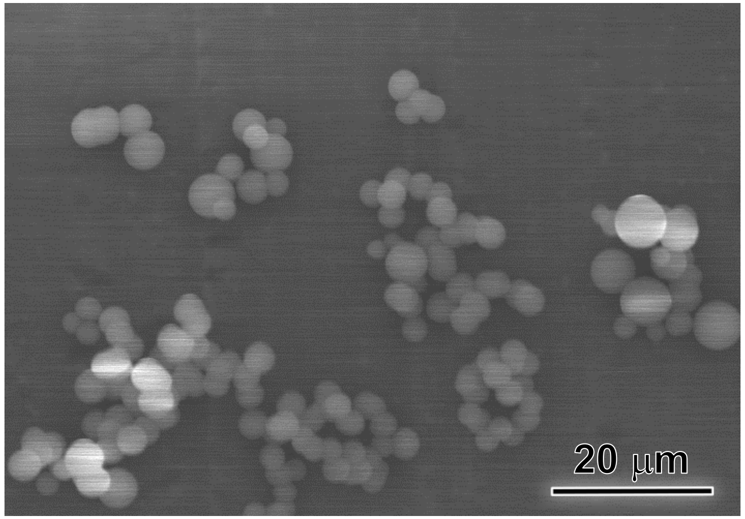

pH 1.7 (prepared with $(COOH)_2$)

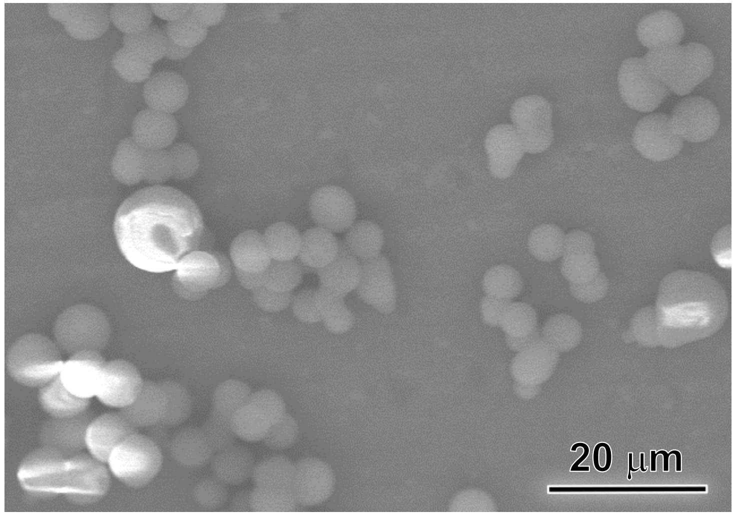

Supplementary Fig. 5 | SEM images of RF-NH_3-523 and the resins prepared at different pH.

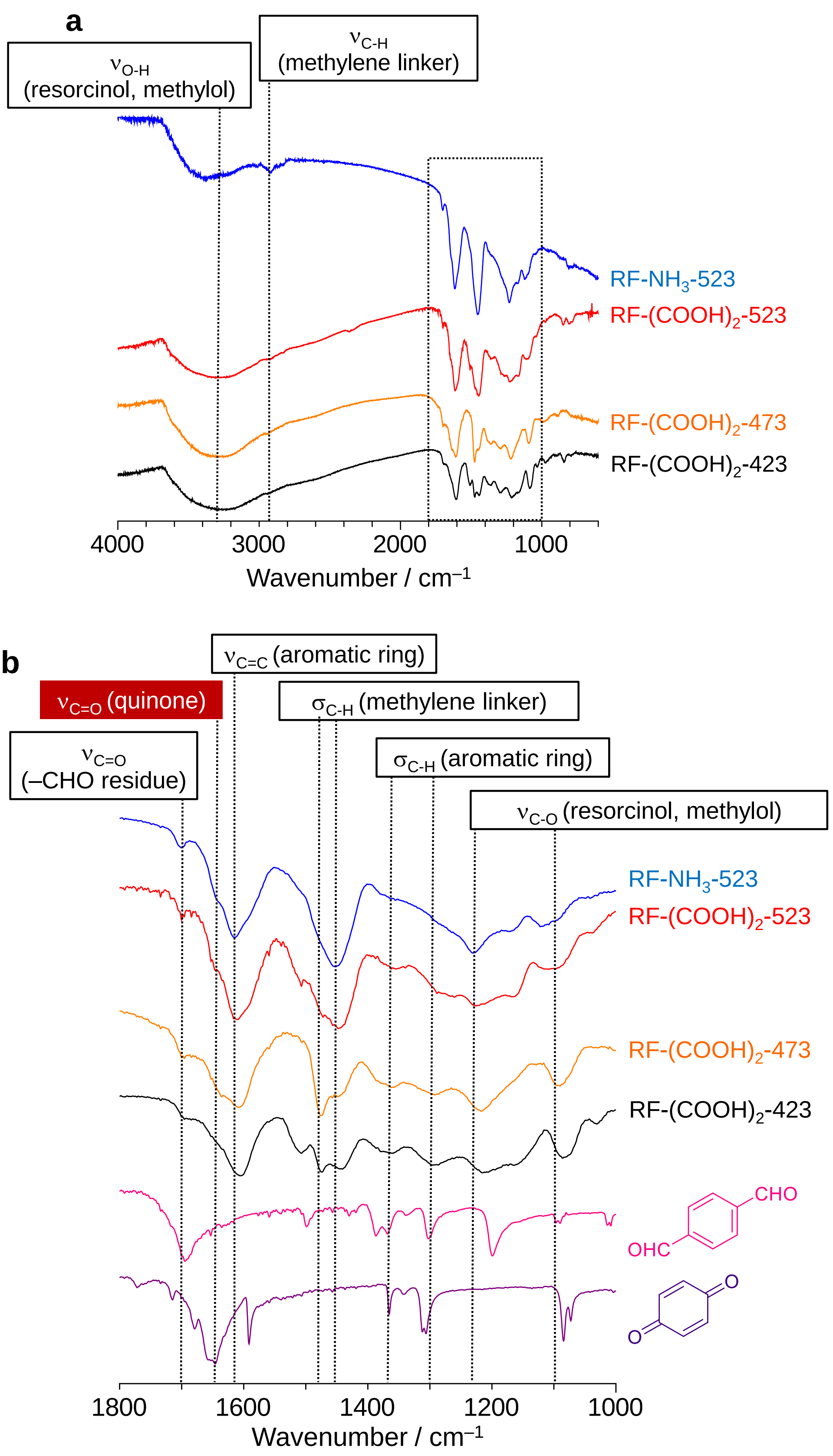

Supplementary Fig. 6 | Fourier-transformed infrared scpectra of RF resins in (a) 1000–4000 cm^{-1} and (b) 1000–1800 cm^{-1} regions. Peak assignments: 3300 cm^{-1} ($\nu_{O\text{–}H}$ of resorcinol or methylol), 2925 cm^{-1} ($\nu_{C\text{-}H}$ of methylene linker), 1700 cm^{-1} ($\nu_{C=O}$ of –CHO residue), 1650 cm^{-1} ($\nu_{C=O}$ of quinone), 1620 cm^{-1} ($\nu_{C=C}$ of aromatic ring), 1490, 1450 cm^{-1} ($\sigma_{C\text{-}H}$ of methylene linker), 1370, 1300 cm^{-1} ($\sigma_{C\text{-}H}$ of aromatic ring), 1240, 1100 cm^{-1} ($\nu_{C\text{-}O}$ of resorcinol and methylol).

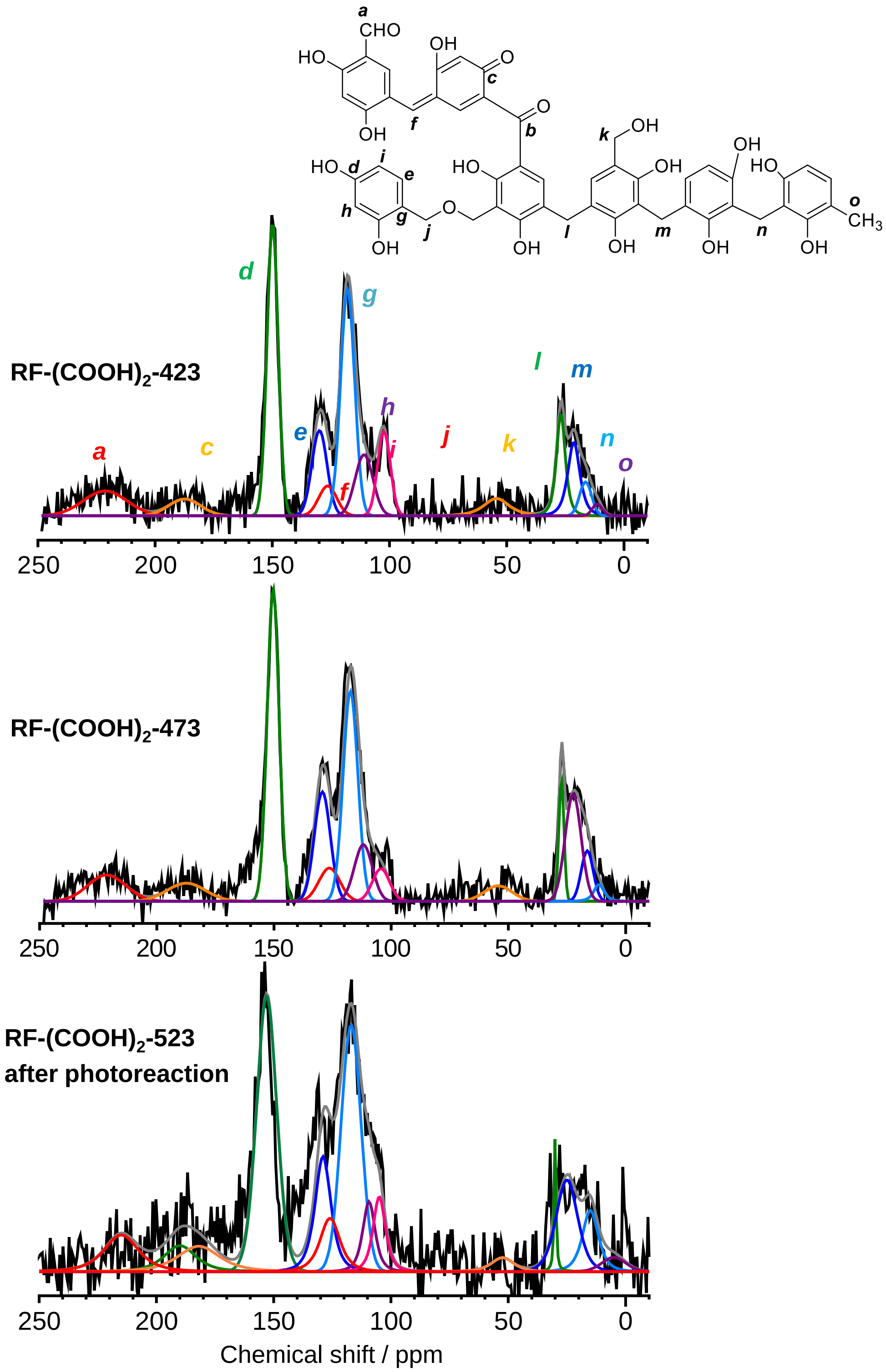

Supplementary Fig. 7 | Solid-state DD/MAS/^{13}C NMR charts of the fresh resins and the RF-(COOH)$_2$-523 resin recovered after photoreaction for 5 h by a solar simulator (Fig. 4d, red). The assignments of the respective carbon components: aldehyde –*C*HO (210 ppm: ***a***), ketone *C*=O (190 ppm: ***b***), quinone *C*=O (182 ppm: ***c***), resorcinol *C*–OH (153 ppm: ***d***), nonsubstituted resorcinol *C* at the *meta* position (130 ppm: ***e***), methine linker –*C*═ (126 ppm: ***f***), substituted resorcinol *C* (117 ppm: ***g***), nonsubstituted resorcinol *C* at the *para* and *ortho* positions (110 ppm: ***h***, 105 ppm: ***i***), methylene ether linker –*C*–O–*C*– (70 ppm: ***j***), methylol *C*–OH (55 ppm: ***k***), methylene linker –*C*– substituted to 4,4′-, 2,4′-, and 2,2′-positions of resorcinols (30 ppm: ***l***, 20 ppm: ***m***, 10 ppm: ***n***), and methyl –*C*H$_3$ (5 ppm: ***o***).

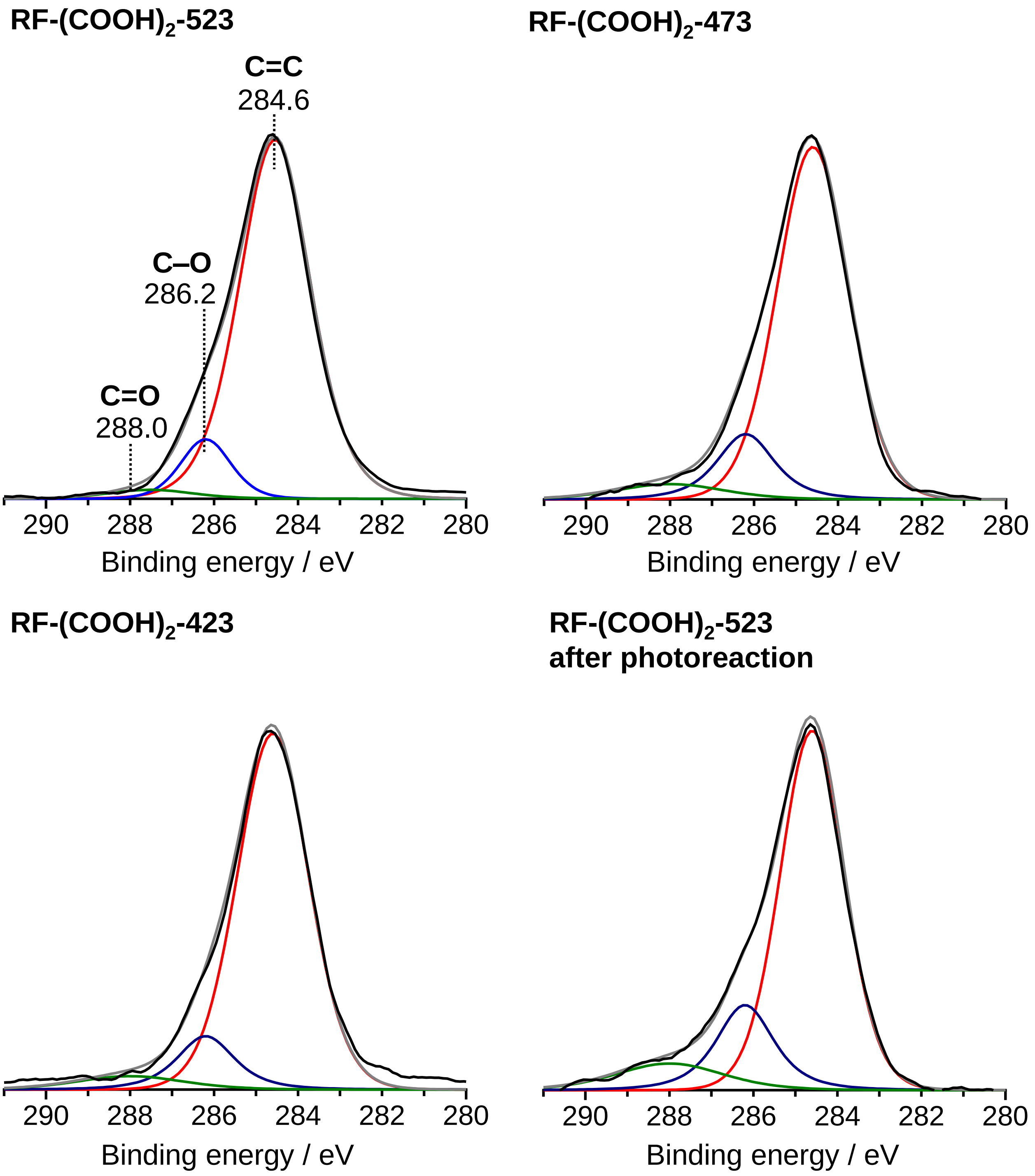

Supplementary Fig. 8 | XPS charts of RF-$(COOH)_2$-523 resin at the C 1s level. Black line is the observed chart, and gray line is sum of the components.

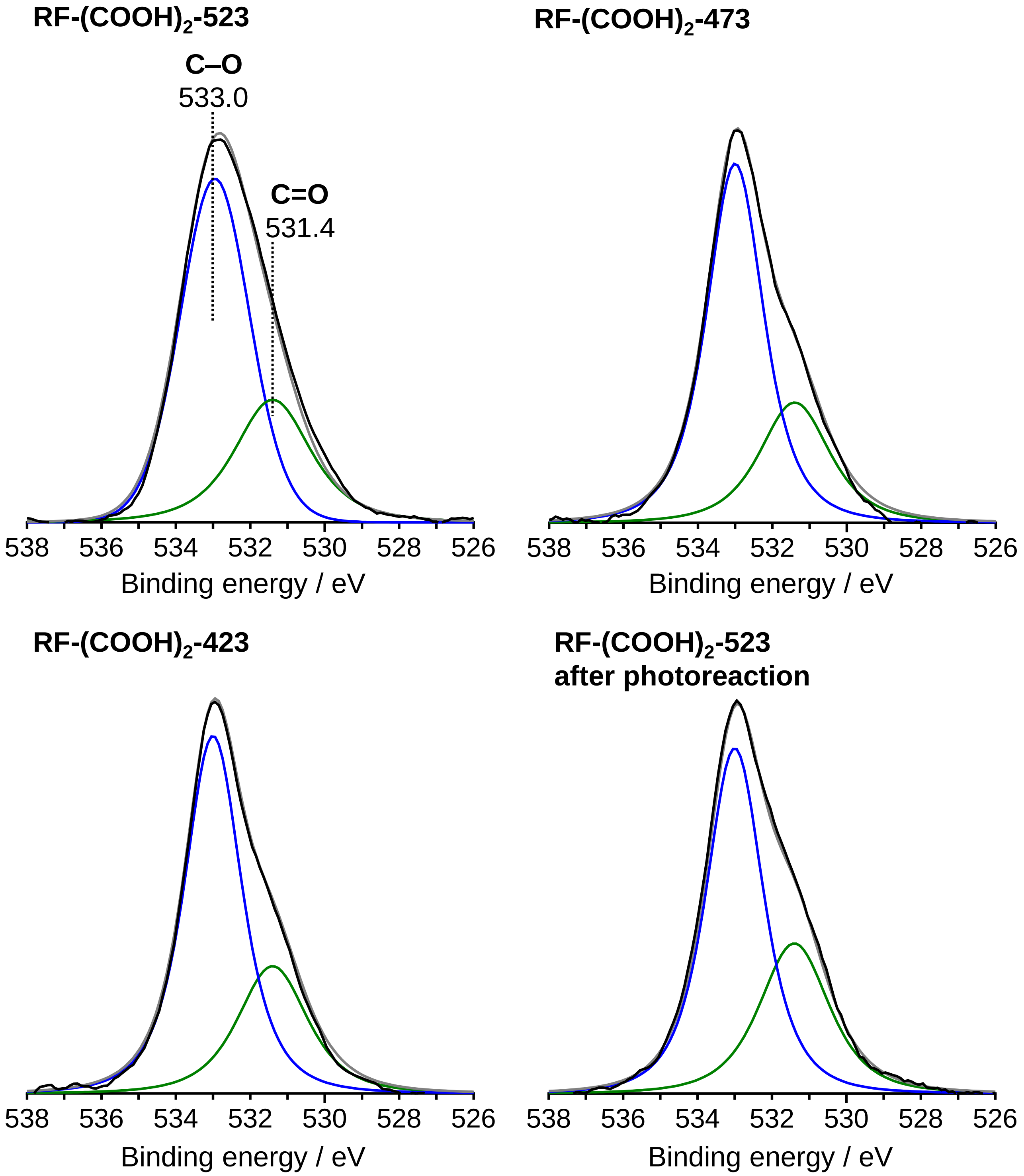

Supplementary Fig. 9 | XPS charts of RF-(COOH)$_2$-523 resin at the O 1s level. Black line is the observed chart, and gray line is sum of the components.

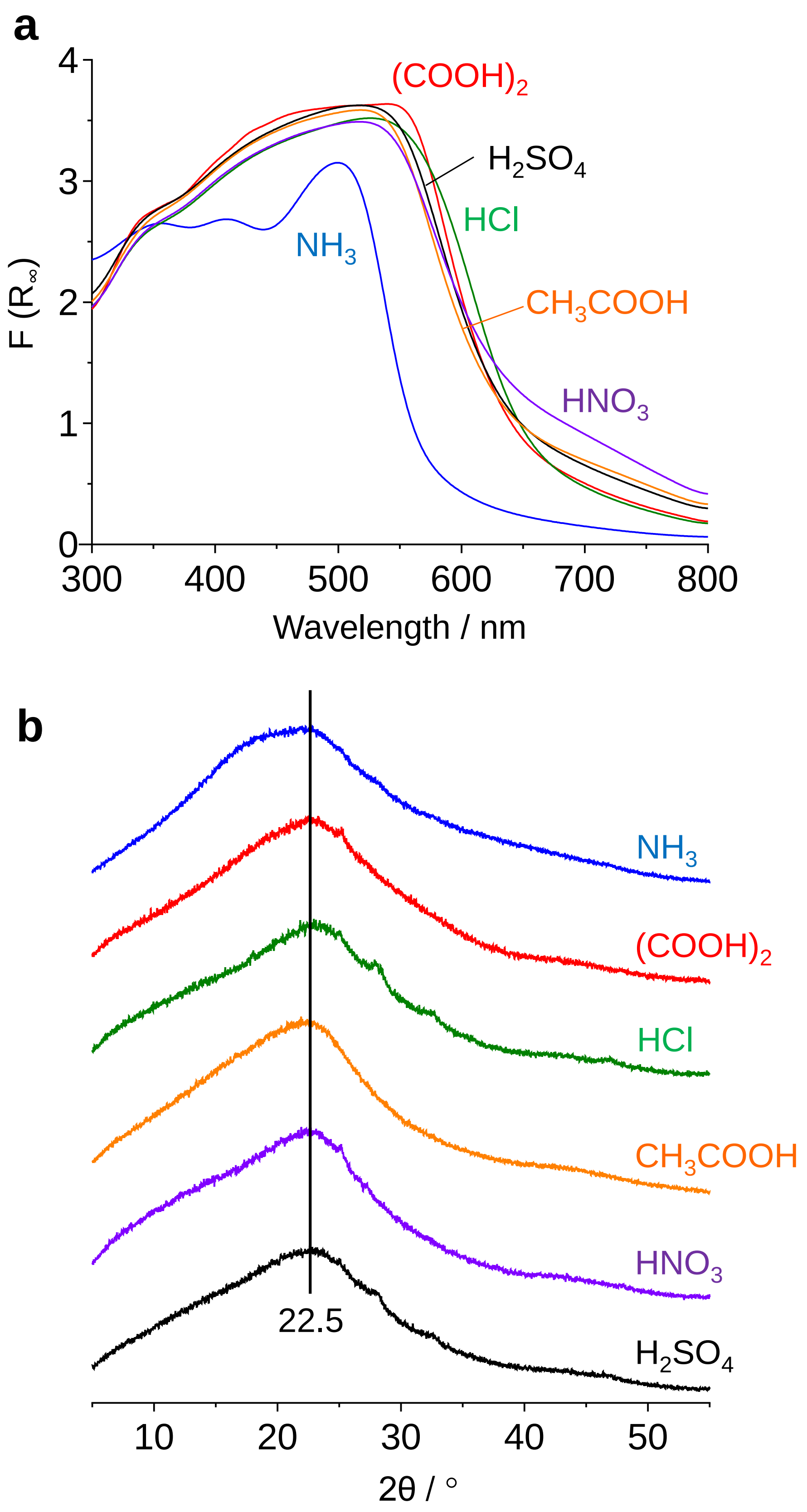

Supplementary Fig. 10 | DR UV-vis and XRD patterns of RF-NH_3-523 and RF-acid-523 resins.

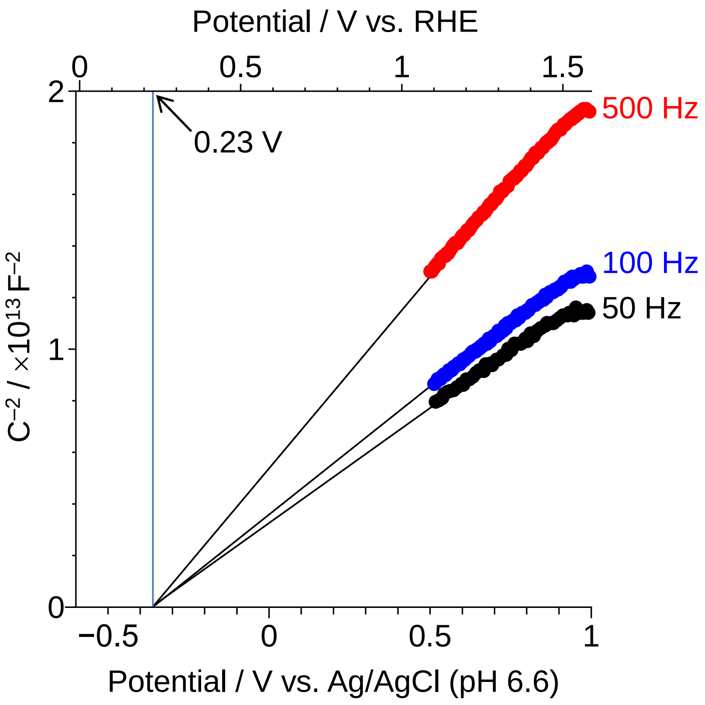

Supplementary Fig. 11 | Electrochemical Mott-Schottky plots of RF-(COOH)$_2$-523. The measurements were performed in 0.1 M Na_2SO_4 solution (pH 6.6). The perturbation signal for the analysis was set at 10 mV.

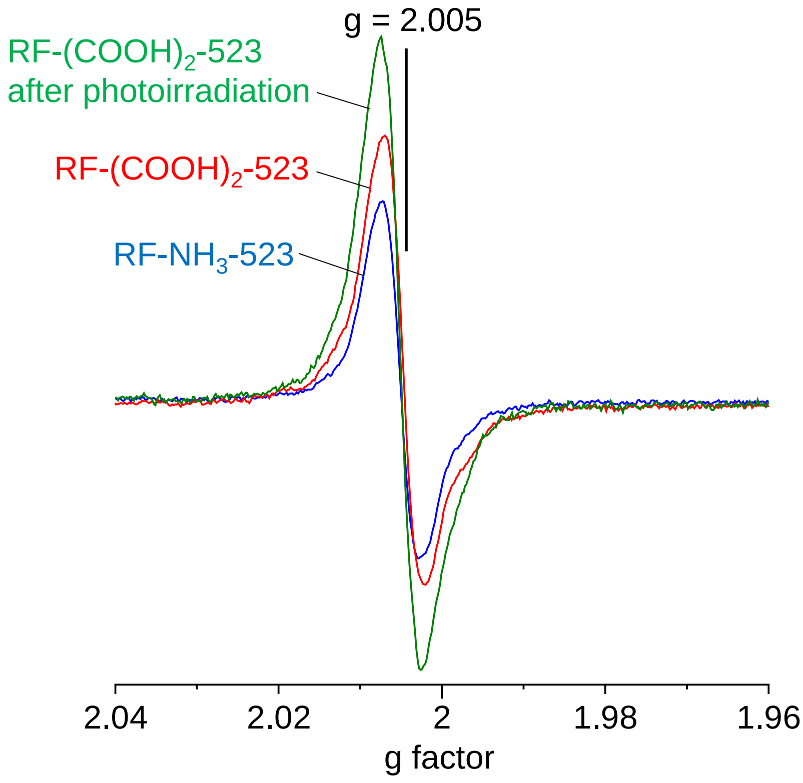

Supplementary Fig. 12 | ESR spectra of the resins. The catalyst (20 mg) was placed in a quartz tube, evacuated at 373 K for 6 h, and cooled to room temperature. The tube was then subjected to analysis at room temperature. Visible light was irradiated to the tube for 1 min at room temperature, and the tube was subjected to analysis.

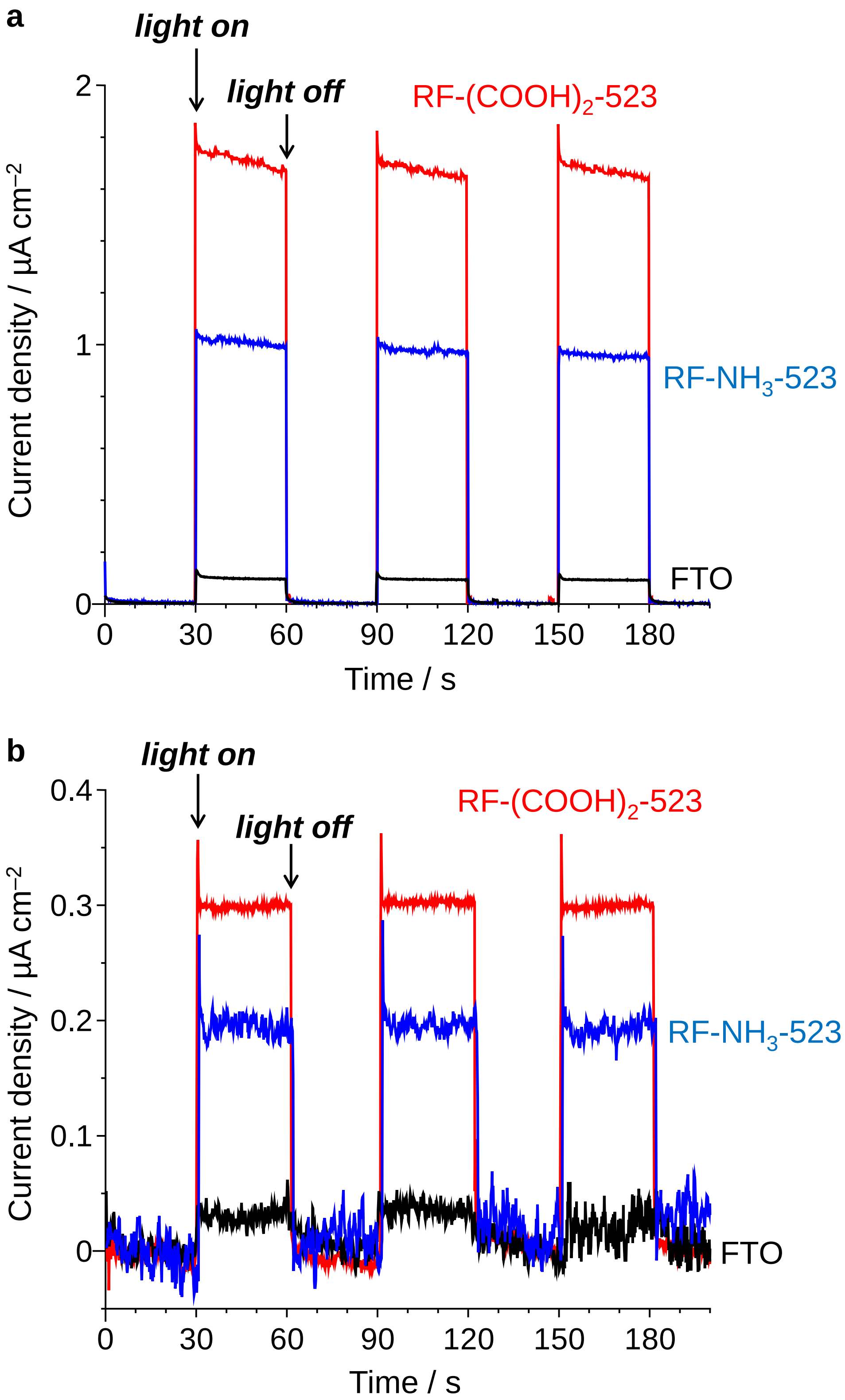

Supplementary Fig. 13 | Photocurrent response of the resin monitored under (a) λ >400 nm and (b) λ = 550 nm monochromatic light irradiation. The measurements were performed on a FTO electrode in 0.1 M Na_2SO_4 solution under visible light at a bias of 0.5 V (vs Ag/AgCl).

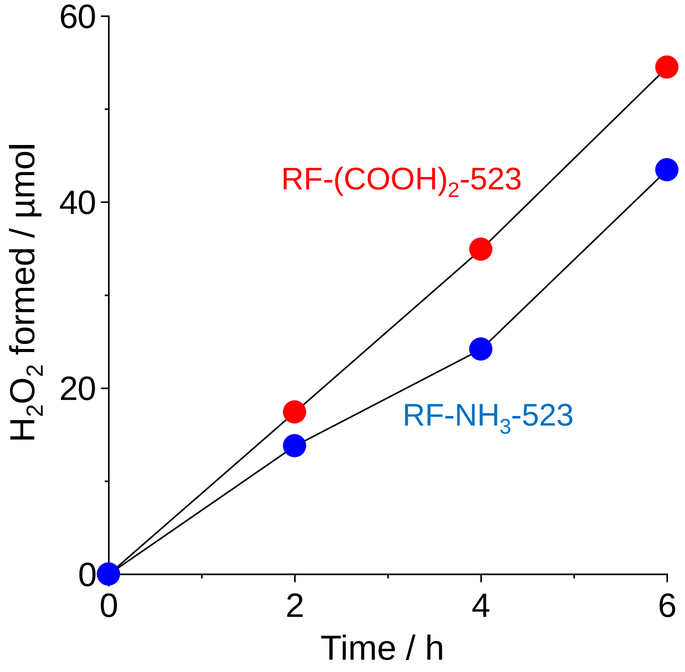

Supplementary Fig. 14 | Amount of H_2O_2 formed on the respective resins during the half photoreaction. Conditions: water (30 mL), resin (50 mg), benzyl alcohol (350 mM), O_2 (1 bar), λ >420 nm (Xe lamp, light intensity at 420–700 nm: 140.3 W m^{-2}), 298 K.

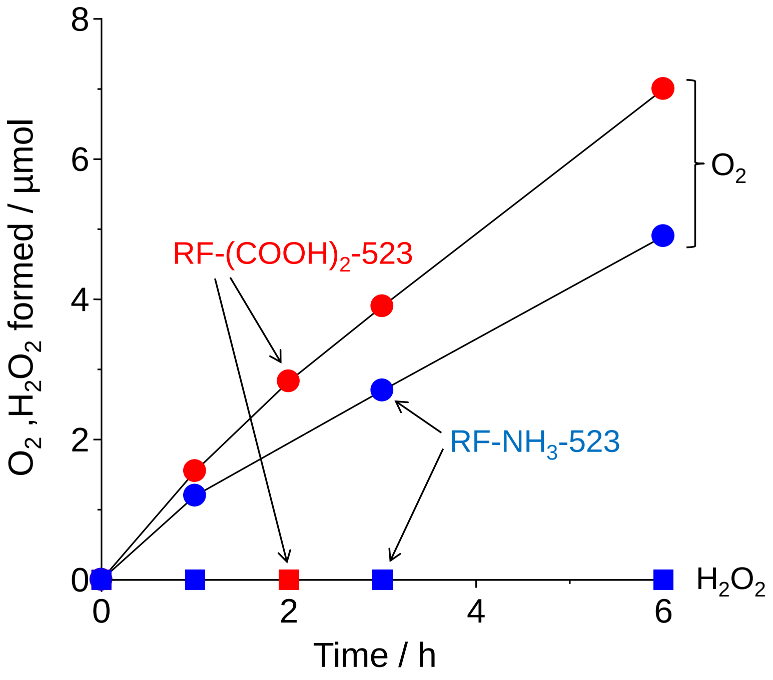

Supplementary Fig. 15 | Amounts of O_2 or H_2O_2 formed on the respective resins during the half photoreaction. Conditions: water (30 mL), RF523 catalyst (50 mg), $NaIO_3$ (2.5 mM), Ar (1 bar), λ >420 nm (Xe lamp, light intensity at 420–700 nm: 140.3 W m^{-2}), 298 K.

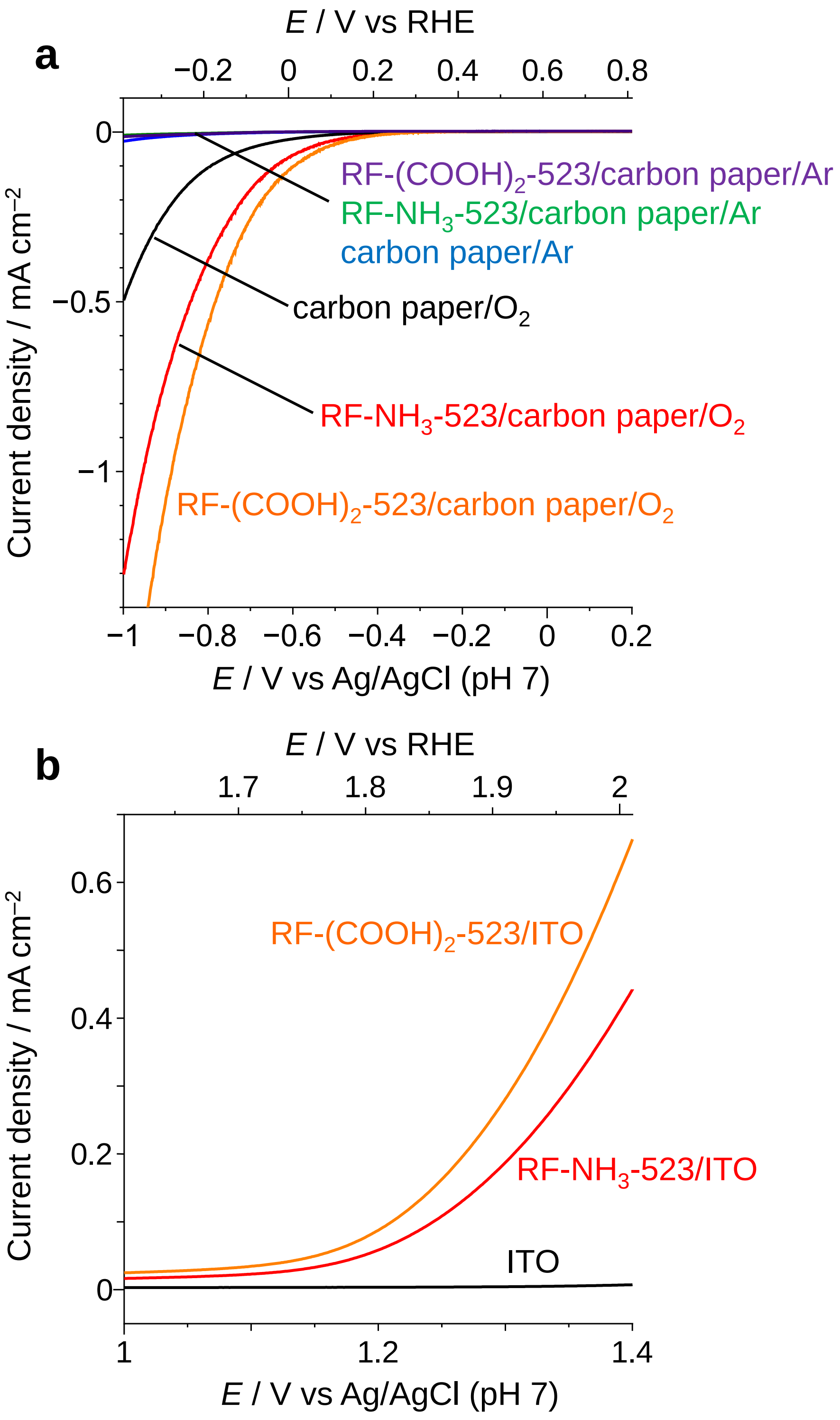

Supplementary Fig. 16 | Linear sweep voltammograms of the respective resins measured on (a) carbon paper and (b) indium tin oxide (ITO). The measurements were carried out in saturated 0.5 M phosphate buffer solution (pH 7) with scan rate of 10 mV s^{-1}.

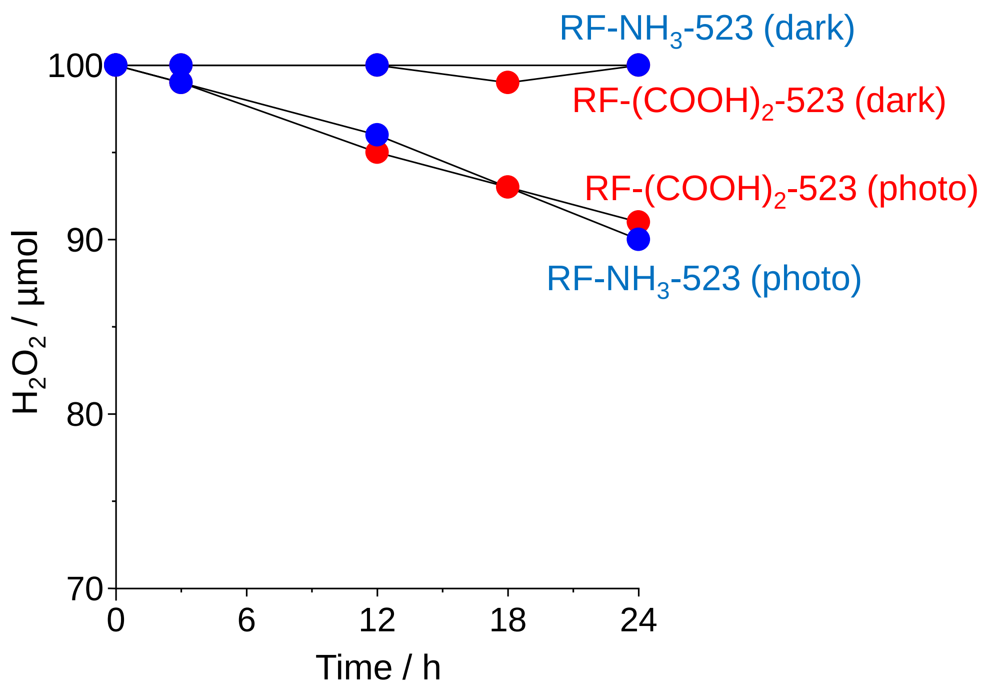

Supplementary Fig. 17 | Change in the H_2O_2 amounts during stirring (red) RF-$(COOH)_2$-523 or (blue) RF-NH_3-523 catalyst in a H_2O_2 solution with $NaIO_3$ as a sacrificial electron acceptor under the dark or photoirradiation condition. Conditions: water (30 mL), H_2O_2 (100 μmol), catalyst (50 mg), $NaIO_3$ (2.5 mM), Ar (1 bar), λ >420 nm (Xe lamp, light intensity at 420−700 nm: 140.3 W m^{-2}), 298 K.

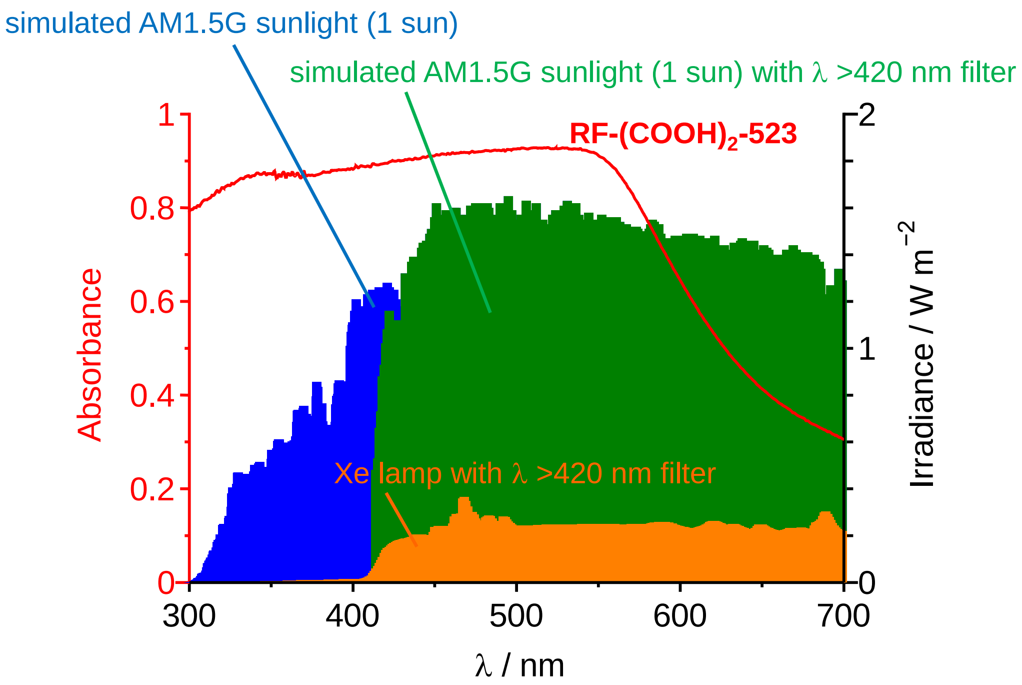

Supplementary Fig. 18 | Absorption spectrum of the RF-$(COOH)_2$-523 resin and light emission spectra for Xe lamp with λ >420 nm filter and AM1.5G simulated sunlight (1 sun) with or without λ >420 nm filter.

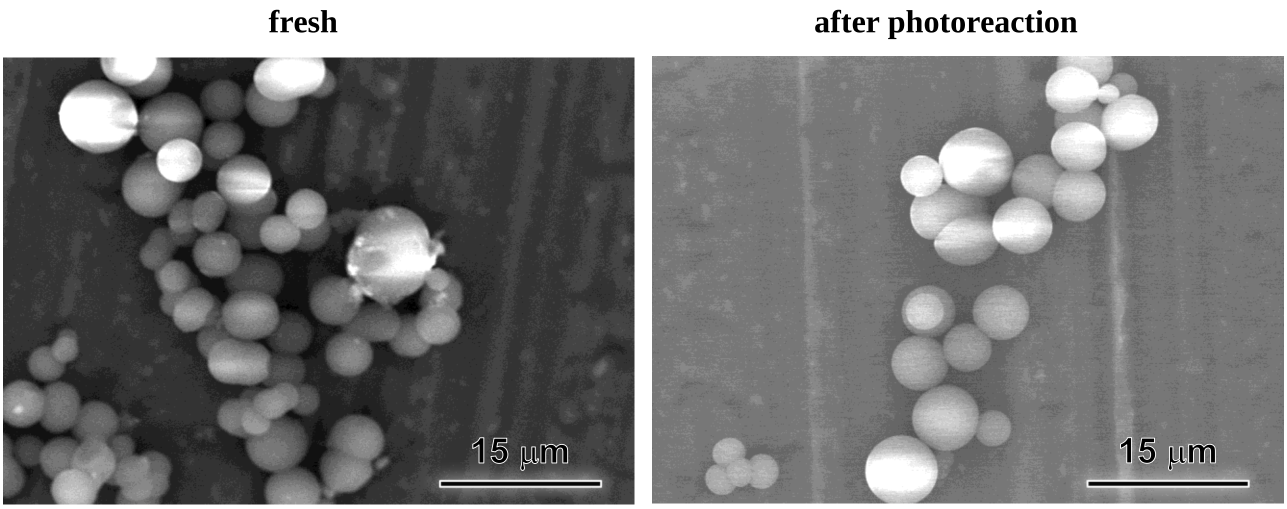

Supplementary Fig. 19 | SEM images of fresh RF-$(COOH)_2$-523 resin and the resin recovered after photoreaction for 5 h by a solar simulator (Fig. 4d).

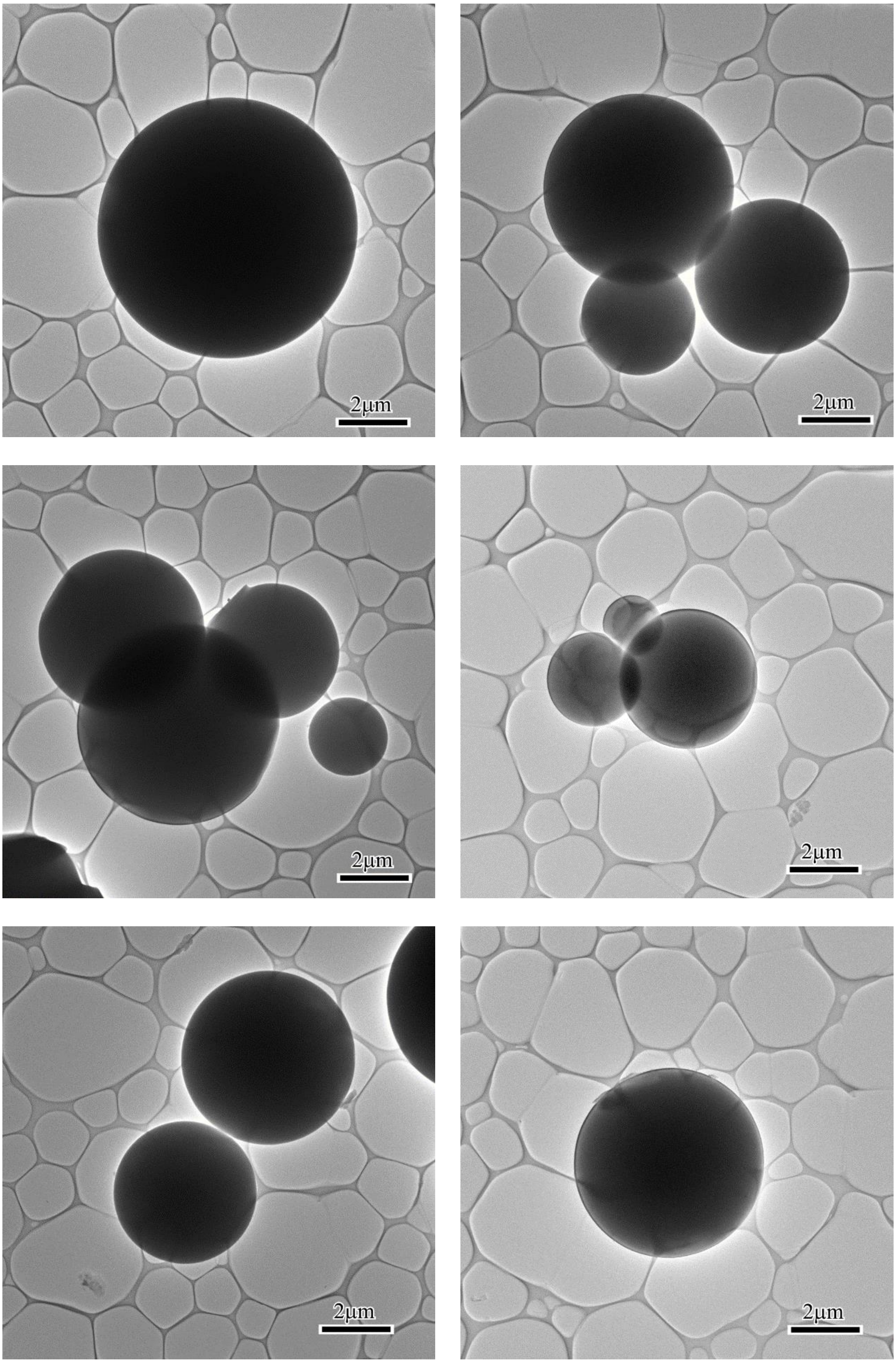

Supplementary Fig. 20 | TEM images of the RF-$(COOH)_2$-523 resin recovered after photoreaction for 5 h by a solar simulator (Fig. 4d).

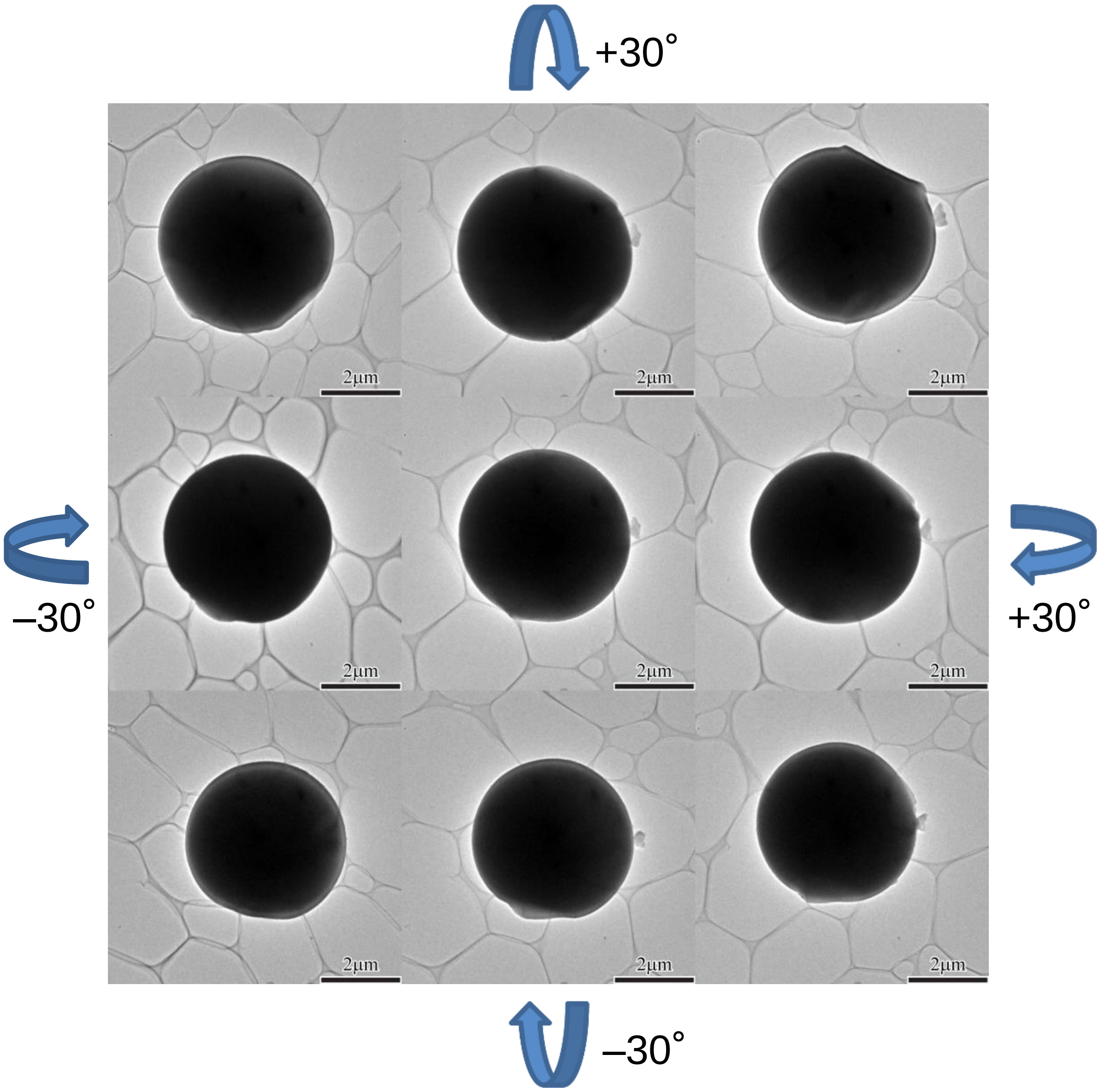

Supplementary Fig. 21 | TEM images of a RF-(COOH)$_2$-523 resin particle recovered after photoreaction for 5 h by a solar simulator (Fig. 4d), observed at different angles.
